# Metabolic changes in glioblastomas in response to choline kinase inhibition: In vivo MRS in rodent models

**DOI:** 10.1002/nbm.4855

**Published:** 2022-11-10

**Authors:** Sourav Bhaduri, Claire Louise Kelly, Clémentine Lesbats, Jack Sharkey, Lorenzo Ressel, Soham Mukherjee, Mark David Platt, Edward J. Delikatny, Harish Poptani

**Affiliations:** ^1^ Centre for Preclinical Imaging, Department of Molecular and Clinical Cancer Medicine University of Liverpool Liverpool UK; ^2^ Division of Radiotherapy and Imaging The Institute of Cancer Research London UK; ^3^ Department of Veterinary Anatomy Physiology and Pathology University of Liverpool Chester UK; ^4^ Department of Radiology, Perelman School of Medicine University of Pennsylvania Philadelphia Pennsylvania USA

**Keywords:** ^1^H MRS, animal model, choline kinase, glioblastoma, JAS239

## Abstract

Changes in glioblastoma (GBM) metabolism was investigated in response to JAS239, a choline kinase inhibitor, using MRS. In addition to the inhibition of phosphocholine synthesis, we investigated changes in other key metabolic pathways associated with GBM progression and treatment response. Three syngeneic rodent models of GBM were used: F98 (*N* = 12) and 9L (*N* = 8) models in rats and GL261 (*N* = 10) in mice. Rodents were intracranially injected with GBM cells in the right cortex and tumor growth was monitored using *T*_2_‐weighted images. Animals were treated once daily with intraperitoneal injections of 4 mg/kg JAS239 (F98 rats, *n* = 6; 9L rats, *n* = 6; GL261 mice, *n* = 5) or saline (control group, F98 rats, *n* = 6; 9L rats, *n* = 2; GL261 mice, *n* = 5) for five consecutive days. Single voxel spectra were acquired on Days 0 (T0, baseline) and 6 (T6, end of treatment) from the tumor as well as the contralateral normal brain using a PRESS sequence. Changes in metabolite ratios (tCho/tCr, tCho/NAA, mI/tCr, Glx/tCr and (Lip + Lac)/Cr) were used to assess metabolic pathway alterations in response to JAS239. Tumor growth arrest was noted in all models in response to JAS239 treatment compared with saline‐treated animals, with a significant reduction (*p* < 0.05) in the F98 model. A reduction in tCho/tCr was observed with JAS239 treatment in all GBM models, indicating reduced phospholipid metabolism, with the highest reduction in 9L followed by GL261 and F98 tumors. A significant reduction (*p* < 0.05) in the tCho/NAA ratio was observed in the 9L model. A significant reduction in mI/tCr (*p* < 0.05) was found in JAS239‐treated F98 tumors compared with the saline‐treated animals. A non‐significant trend of reduction in Glx/tCr was observed only in F98 and 9L tumors. JAS239‐treated F98 tumors also showed a significant increase in Lip + Lac (*p* < 0.05), indicating increased cell death. This study demonstrated the utility of MRS in assessing metabolic changes in GBM in response to choline kinase inhibition.

## Graphical abstract

Changes in glioblastoma (GBM) metabolism were investigated in response to JAS239, a choline kinase inhibitor, using MRS in three syngeneic rodent models of GBM. Tumor growth arrest was noted in all models in response to JAS239 treatment compared with saline‐treated animals. A significant reduction in the tCho/NAA ratio was observed with treatment in the 9L model; a significant reduction in mI/tCr was found with treatment in F98 tumors along with significant increase in Lip + Lac with treatment, indicating increased cell death.

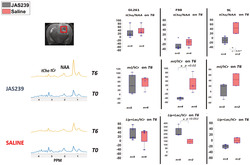

**Abbreviations**
ChoK: choline kinase
CRLB: Cramér–Rao lower bound
GBM: glioblastoma multiforme
Glx: glutamate–glutamine
Gly: glycine
GS: glutamine synthetase
HPF: high‐power field
Lip + Lac: lipid–lactate
mI: myo‐inositol
NAA: *N*‐acetylaspartate
PC: phosphatidylcholine
PRESS: point resolved spectroscopy
PUFA: polyunsaturated fatty acid
TCA: tricarboxylic acid
tCho: total choline
tCr: total creatine
*T*_E_
: echo time
*T*_R_
: repetition time

## 1 INTRODUCTION

Glioblastoma multiforme (GBM) is the most aggressive and fast‐growing primary brain tumor, with a median survival rate of only 14–16 months after diagnosis and a 5 year survival rate of less than 5%, rendering GBM the deadliest brain tumor in adults.^1^ The current standard of care is inadequate in curing patients due to the highly heterogeneous and infiltrating nature of GBM. Tumor recurrence is inevitable, even if maximal resection is achieved. Therefore, novel treatment strategies have focused on targeting specific pathways involved in tumor progression and invasion, amongst other key characteristics, to improve treatment response.^2^

Aberrant cellular metabolism is a widely accepted hallmark of cancer, and the most commonly recognized metabolic shift in tumor cells is aerobic glycolysis.^3^ However, metabolic flexibility has been demonstrated within highly heterogeneous tumors such as GBM.^4^, ^5^ For example, brain tumor patients infused with ^13^C‐glucose during resection surgery demonstrated ^13^C‐pyruvate oxidation by pyruvate dehydrogenase feeding into the TCA cycle,^4^ possibly as a mechanism to adapt to the ever‐changing tumor microenvironment. Therefore, targeting metabolism in general, as opposed to specific molecular targets, is an extremely attractive approach for novel anti‐cancer therapies, since many oncogenic phenotypes are attributed to metabolic reprogramming. For instance, alterations in PDGFRA, EGFR and NF1 genes, which have been shown to influence metabolism in numerous ways, have been identified in the molecular subgroups of GBM. EGFR expression has been associated with a reduction in membrane permeability to total choline (tCho).^6^ Targeting metabolism is a potential strategy for overcoming treatment resistance.^7^

MRS is a non‐invasive technique that enables the quantification of metabolites in real time^8^, ^9^ and therefore has significant potential as a complementary method to MRI in grading human brain tumors and monitoring therapy response.^10^, ^11^ Metabolic signatures of tumors classically demonstrate increased tCho and lactate levels compared with healthy brain tissue,^12^ with concomitant reduction in the neurometabolite *N*‐acetylaspartate (NAA).^11^, ^12^, ^13^ Total creatine (tCr) is a marker of the energy status of the cell and has been proposed as an effective prognostic marker as it has been shown to be a predictor of tumor progression; gliomas with reduced tCr tend to have longer progression‐free survival.^10^ Increased lactate levels are indicative of active glycolysis and associated with a hypoxic environment.^14^ Another relevant metabolic marker elevated in GBM is myo‐inositol (mI), an important substrate for the production of phosphatidylinositol lipids.^13^, ^15^ The combination of glutamine and glutamate (Glx) can be measured via MRS. Glutamine is the most abundant circulating amino acid and is essential for cell function such as synthesis of mitochondrial precursors.^16^ Glutamate is the main excitatory neurotransmitter, regulating brain function and development. Under the process termed ‘glutaminolysis’, glutamine is converted to glutamate via glutaminase enzymes. Glutamate then undergoes reduction into α‐ketoglutarate, a key intermediate in the tricarboxylic acid (TCA) cycle. Therefore, it is not surprising that alterations in Glx are associated with high‐grade GBM.^17^, ^18^, ^19^

Inhibiting choline kinase (ChoK) α has shown promising results in vivo across rodent models of breast cancer and GBM.^20^, ^21^ The first ChoK inhibitor, hemicholinium‐3 (HC‐3), demonstrated promising results in vitro; however, its in vivo potency was poor and induced off target toxicity in mice.^22^

Further, a study assessing a more promising ChoK inhibitor, MN58b, demonstrated that ^1^H MRS can be used to detect a decrease in tCho as a pharmacokinetic marker for treatment response and inhibition of ChoK activity by MN58b in a rat model of GBM.^20^ A second‐generation ChoK inhibitor, JAS239, with near‐infrared fluorescent properties, has been reported to be a potent inhibitor of ChoK in mouse models of breast cancer.^21^ Therefore, this study was performed to investigate the effect of JAS239 on rodent GBM metabolism using MRS.

## 2 MATERIALS AND METHODS

To test the generalizability of our approach, three syngeneic rodent models of GBM were used in this study: the F98 and 9L GBM models in rats and the GL261 model in mice. These models recapitulate several features of human GBM and have been used previously in the MRI literature.^23^, ^24^, ^25^, ^26^ Animal studies were conducted in compliance with the UK Home Office Animals (Scientific Procedures) Act 1986 and with ethical approval from the local animal welfare committee.

F344 female Fischer rats (weight 100–120 g) were implanted with 50 000 F98 cells (ATCC CRL‐2937™) (*N* = 12) or 100 000 9L cells (ATCC CRL‐2200™) (*N* = 8) whilst C57BL6 male mice (age 8–10 weeks, weight 20–25 g, *N* = 10) were implanted with 500 000 GL261 cells (ATCC, Manassas, VA, USA). Intracranial tumors were developed by transcranial injection of GBM cells into the right cortex. Animals were anesthetized with 2% isoflurane in O_2_ and the respiration rate and body temperature were monitored using an abdominal motion sensor and a rectal probe. Then they were secured on a stereotaxic frame, a burr hole was drilled through the skull 3 mm right for rats (1.5 mm right for mice) and 3 mm posterior for rats (2 mm posterior for mice) from the bregma, and GBM cells suspended in 5 μL of serum‐free medium were injected 2.5 mm into the brain cortex for rats (2 mm for mice).

Imaging was performed using a 9.4 T Bruker BioSpec scanner (Bruker BioSpin, Ettlingen, Germany). Signal was generated using an 86 mm transmission birdcage coil and detected by a four‐channel phased array surface coil. Animals were anesthetized as described above. *T*_2_‐weighted images using a RARE (rapid acquisition with relaxation enhancement) sequence (*T*_R_/effective *T*_E_ 4167/33 ms, 0.3 mm slice thickness, field of view (FOV) = 30 × 30 mm^2^, 256 × 256 matrix, RARE factor = 8) were acquired. Once the tumors were more than 3 mm in diameter, typically 8–10 days after implantation for GL261 tumors and F98 tumors and 18–20 days for 9L tumors, longitudinal MRS studies were performed for therapeutic monitoring in response to JAS239 treatment (4 mg/kg/day injected intraperitoneally for five consecutive days) in F98 rats, *n* = 6; 9L rats, *n* = 6; GL261 mice, *n* = 5. Control animals received saline injections (F98 rats, *n* = 6; 9L rats, *n* = 2; GL261 mice, *n* = 5). Animals were imaged on Day 0 (T0, baseline) and Day 6 (T6, end) of treatment. Tumor volumes were calculated by multiplying the number of tumor pixels in the region of interest (ROI) by the pixel dimensions. Single voxel (2 × 2 × 2 mm^3^) spectra were acquired using a point resolved spectroscopy (PRESS) sequence from the tumor as well as the contralateral hemisphere covering a region beyond the midline so that it is completely in a tumor‐free region of the brain, excluding ventricles (defined by *T*_2_‐weighted imaging) and accounting for any tumor‐induced midline shift. Whilst it is not truly contralateral to the tumor voxel, it provides the best alternative without confounding contamination from ventricle, scalp or shimming issues. The following sequence parameters were used: *T*_R_ = 2500 ms for rat data (2000 ms for mice), *T*_E1_ = 9.13 ms and *T*_E2_ = 7.37 ms, number of averages = 256 for rat data studies (150 for mice data experiments), complex points = 2048 and spectral width = 4401 Hz. Fieldmap based shimming (Mapshim) was used.

Prior to quantifying the water suppressed spectroscopy data, an HLSVD (Hankel–Lanczos singular value decomposition) filter with a model order of 25 was used to suppress the residual water peak at 4.7 ppm.^27^, ^28^ Apodization was performed with a 5 Hz Lorentzian function and zero filling by a factor of two. All these steps were performed using jMRUI software.^29^ Metabolite peaks were fitted using the QUEST (quantum estimation) algorithm^30^, ^31^ within jMRUI. A basis set of the metabolites lipid–lactate (Lip + Lac) at 1.3 ppm, NAA at 2.01 ppm, Glx at 2.35 and 3.74 ppm, tCr at 3.03 ppm, tCho at 3.2 ppm, mI + glycine (mI + Gly) at 3.56 ppm and other metabolites including phosphocreatine (at 3.9 ppm), lipid (at 0.9 ppm), aspartate, acetate, alanine, GABA, glucose, glycerophosphocholine, glutathione, N‐acetylaspartylglutamate, phosphorylcholine, scyllo‐inositol and taurine were simulated using the NMR scope tool^32^ inside jMRUI. Water amplitude was calculated from the unsuppressed water spectrum. For scaling the spectra to unsuppressed water signal, the following correction factors were taken into account for the *T*_1_ and *T*_2_ of water.

$$\frac{\left\{1-\exp\left(\frac{-T_{R}}{{T_{1}}_{\text{water}}}\right)\right\}}{\exp\left(\frac{T_{E}}{{T_{2}}_{\text{water}}}\right)}$$

using *T*_1_ = 2097 ± 68 ms and *T*_2_ = 42 ± 1.6 ms as reported for the rat brain.^33^ The Cramér–Rao lower bound (CRLB) criterion was used to evaluate the quality of the fitting. A CRLB/amplitude of less than 20% was selected as the criterion to discriminate well fitted metabolites from poorly fitted ones. All metabolites within a spectrum that met this criterion were used for data analysis and the ones that did not were excluded. The tCho concentration (tCho/water) and ratios (tCho/tCr, tCho/NAA) were then calculated.

After the imaging and MRS experiments, the animals were sacrificed via an overdose of 3 mL/kg pentobarbital sodium (Euthatal, Merial Animal Health, Harlow, UK) injected intraperitoneally. An incision was made along the mid‐ventral line through the abdomen, and the aorta under the diaphragm was cut. A 25‐gauge needle connected to an extension tube was clamped to the left ventricle. 50 mL of PBS followed by 75 mL of 4% formalin (Sigma‐Aldrich, St. Louis, MO, USA) was perfused through the heart. Following fixation, brains were collected and suspended in 4% formalin. Brains were cut into approximately 2 mm transverse sections at the tumor site and embedded in paraffin. Histological sections 4  μm thick containing tumors and surrounding brain tissue were obtained and stained with hematoxylin and eosin. The mitotic index was evaluated at the interface between the neoplasm and the surrounding brain (infiltrating margin), quantified as the average of the number of mitotic figures detected per high‐power field (HPF: 400× magnification) over a total of five tumor margins representing randomly selected HPFs per tumor.

### 2.1 Statistical analysis

Significance level was set at *p* ≤ 0.05. Differences between the JAS239 and control group (% change in tumor volume and metabolite ratios on T6 scans with respect to T0) were evaluated using the Wilcoxon rank‐sum test. The Bonferroni adjustment was performed to adjust *p* by the total number of comparisons being carried out. All analyses were conducted using MATLAB software.

### 2.2 Data access statement

Research data supporting this publication are available at https://doi.org/10.17638/datacat.liverpool.ac.uk/1719.

## 3 RESULTS

**FIGURE 1:**
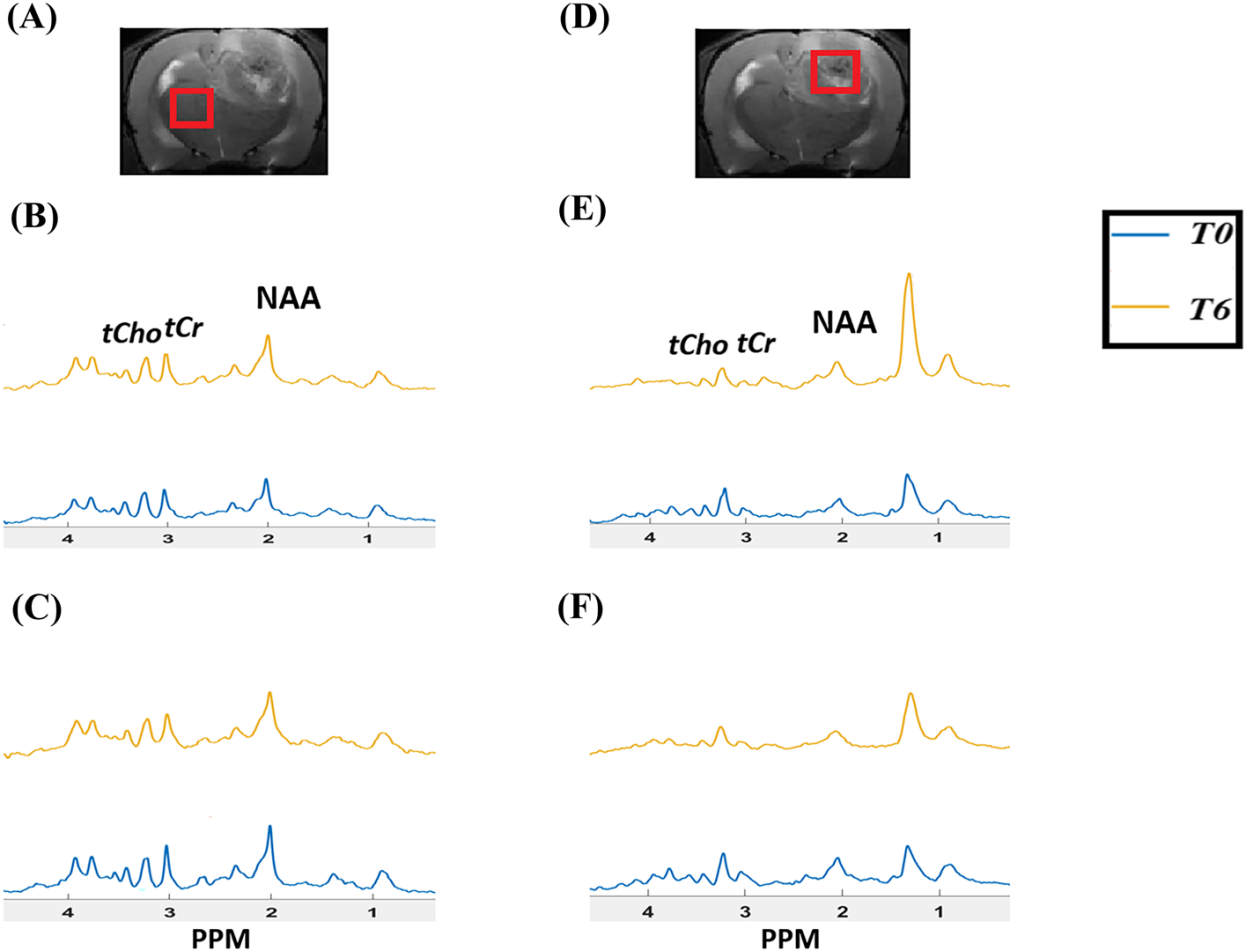
A, D, Representative *T*_2_‐weighted images from F98 rat GBM showing voxel placement in contralateral region (A) and tumor (D). B, C, ^1^H MR spectra of contralateral region showing effect of JAS239 on tCho (B) compared with saline (C) treatment at different time points. E, F, ^1^H MR spectra of tumor region showing effect of JAS239 on Cho (E) compared with saline treatment (F). The unsuppressed water signal was used to scale the spectra.

*T*_2_‐weighted MR images with voxel placement and in vivo MR spectra of a representative F98 rat GBM are shown in Figure 1A–C for the normal contralateral brain and in Figure 1D–F for the tumor region. Spectra are shown for rats treated with JAS239 (Figure 1B and 1E) and for a control tumor treated with saline (Figure 1C and 1F). The typical metabolic profile of increased tCho (3.2 ppm) and reduced NAA (2.0 ppm) was evident in the tumor spectra (Figure 1E and 1F), and JAS239 treatment induced a reduction in the tCho peak (Figure 1E).

In Table 1, quantitative information is provided on data quality in terms of linewidth of tCr in different groups, SNR and CRLB.

**TABLE 1:** Linewidth of tCr, SNR (measured as ratio between tCr (for contralateral spectra) or tCho (for tumor spectra) peak amplitude and standard deviation of noise) and CRLB of fitted peaks in different groups

	Linewidth (Hz)	SNR (a.u.)	% CRLB (tCr)	% CRLB (tCho)	% CRLB (NAA)	% CRLB (mI)	% CRLB (Glx)	% CRLB (Lip + Lac)
GL261 tumor	9.89 ± 2.0	10.34 ± 1.70	2.22 ± 0.83	2.2 ± 0.79	2.26 ± 0.87	9.81 ± 3.77	9.828 ± 3.77	12.718 ± 3.91
GL261 contralateral	8.48 ± 0.72	9.72 ± 1.92	3.07 ± 0.42	2.97 + 0.55	5.62 ± 1.03	7.61 ± 3.52	9.66 + 0.54	13.23 ± 1.54
F98 tumor	9.79 ± 0.57	13.41 ± 1.64	2.29 ± 0.38	2.33 ± 0.31	4.01 ± 0.78	11.24 ± 2.15	11.39 ± 2.19	14.92 ± 2.74
F98 contralateral	7.68 ± 1.04	11.684 ± 1.72	2.29 ± 0.41	2.27 ± 0.39	4.36 ± 0.67	7.15 ± 1.82	7.18 ± 1.81	9.71 ± 2.44
9L tumor	9.63 ± 0.96	14.91 ± 3.75	2.02 ± 0.28	2.06 ± 0.14	3.07 ± 0.45	8.43 ± 3.30	8.40 ± 3.43	11.36 ± 4.34
9L contralateral	7 ± 0.86	11.4 ± 0.22	2.15 ± 0.18	2.46 ± 0.33	5.26 ± 0.53	9.77 ± 0.40	9.82 ± 0.38	13.02 ± 0.68

**FIGURE 2:**
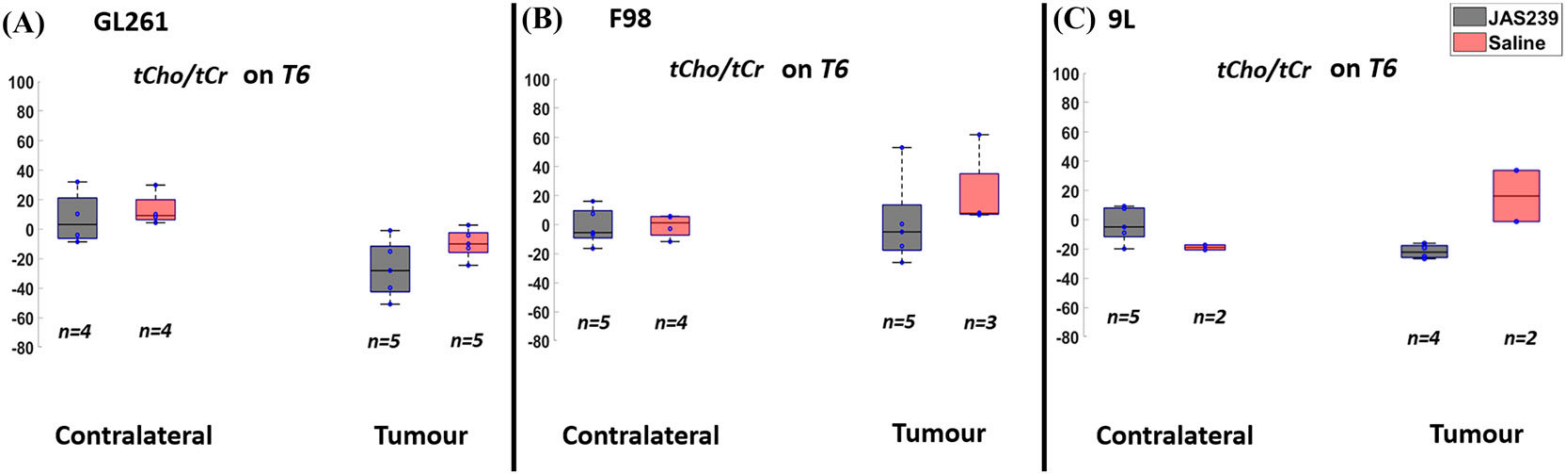
Box plots comparing percentage change (with respect to baseline) in tCho/tCr ratio between JAS239 and control groups in contralateral and tumor regions in GL261 mouse GBM (A), F98 rat GBM (B) and 9L rat GBM (C). *n* denotes the number of samples used for quantification considering the CRLB criteria.

**FIGURE 3:**
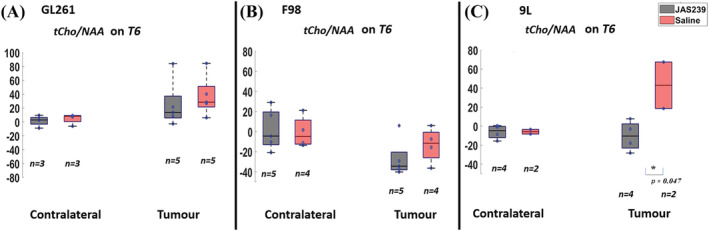
Box plots comparing percentage change (with respect to baseline) in tCho/NAA ratio between JAS239 and control groups in contralateral and tumor regions in GL261 mouse GBM (A), F98 rat GBM (B) and 9L rat GBM (C). A single asterisk indicates that the difference between groups reached a significance level of 0.05. *n* denotes the number of samples used for quantification considering the CRLB criteria.

**FIGURE 4:**
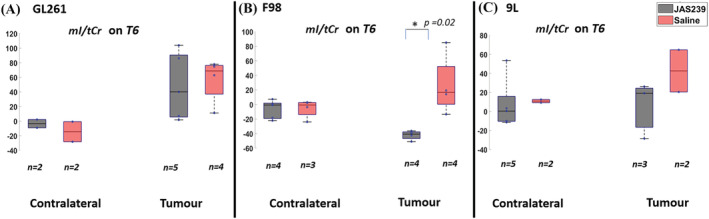
Box plots comparing percentage change (with respect to baseline) in mI/tCr ratio between JAS239 and control groups in contralateral and tumor regions in GL261 mouse GBM (A), F98 rat GBM (B) and 9L rat GBM (C). A single asterisk indicates that the difference between groups reached a significance level of 0.05. *n* denotes the number of samples used for quantification considering the CRLB criteria.

**FIGURE 5:**
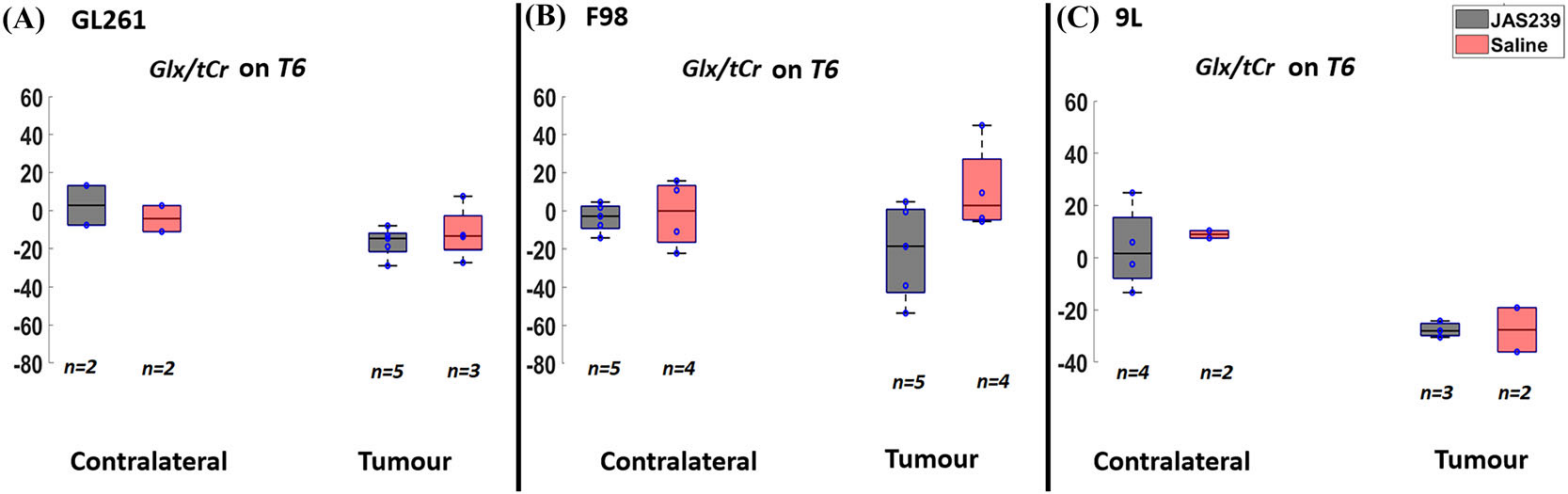
Box plots comparing percentage change (with respect to baseline) in Glx/tCr ratio between JAS239 and control groups in contralateral and tumor regions in GL261 mouse GBM (A), F98 rat GBM (B) and 9L rat GBM (C). *n* denotes the number of samples used for quantification considering the CRLB criteria.

**FIGURE 6:**
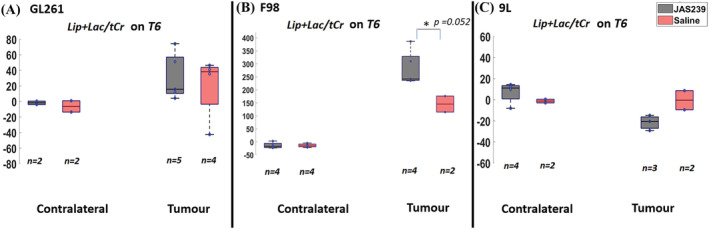
Box plots comparing percentage change (with respect to baseline) in (Lip + Lac)/tCr ratio between JAS239 and control groups in contralateral and tumor regions in GL261 mouse GBM (A), F98 rat GBM (B) and 9L rat GBM (C). A single asterisk indicates that the difference between groups reached a significance level of 0.05. *n* denotes the number of samples used for quantification considering the CRLB criteria.

To determine the effect of JAS239 on GBM metabolism in the three tumor models we calculated the percentage change from baseline of several metabolites, relative to two of the most commonly used reference metabolites, tCr (3.02 ppm, Figure 2 and Figures 4, 5, 6) and the neuronal metabolite NAA (2.0 ppm, Figure 3). The percentage change with respect to the baseline of tCho, mI, Glx and Lip + Lac relative to water between JAS239 and untreated groups in contralateral and tumor regions in GL261 mice, F98 and 9L rat GBMs can be found in Supplementary Figures S1 –S4. Across all models the percentage change of the tumor tCho/tCr ratio was reduced non‐significantly in JAS239‐treated animals (Figure 2A–C) at T6. The largest reduction was evident in 9L tumors (−42%) as compared with saline‐treated rats; however, this was not statistically significant due to variation in 9L control data, which comprised only two animals. These trends were also evident when we assessed percentage change from baseline of the tCho/NAA ratio between treated and untreated groups (Figure 3A–C) at T6; the 9L model demonstrated a significant reduction (−53%; *p* = 0.047) while both GL261 and F98 models demonstrated non‐significant trends of reduction in the tCho/NAA ratio.

In response to JAS239 treatment, F98 tumors demonstrated a significant reduction in the mI/tCr ratio as compared with untreated rats (*p* = 0.02, Figure 4B) at T6 whilst levels from the contralateral regions were comparable; GL261 and 9L treated tumors also exhibited a trend of reduction (Figure 4A and 4C) at T6. In addition, there was a 32% reduction in Glx/tCr in JAS239‐treated F98 tumors as compared with untreated rats (Figure 5A–C) at T6; however, no significant changes in the Glx/tCr ratios was observed in 9L or GL261 tumors in response to JAS239 treatment.

**FIGURE 7:**
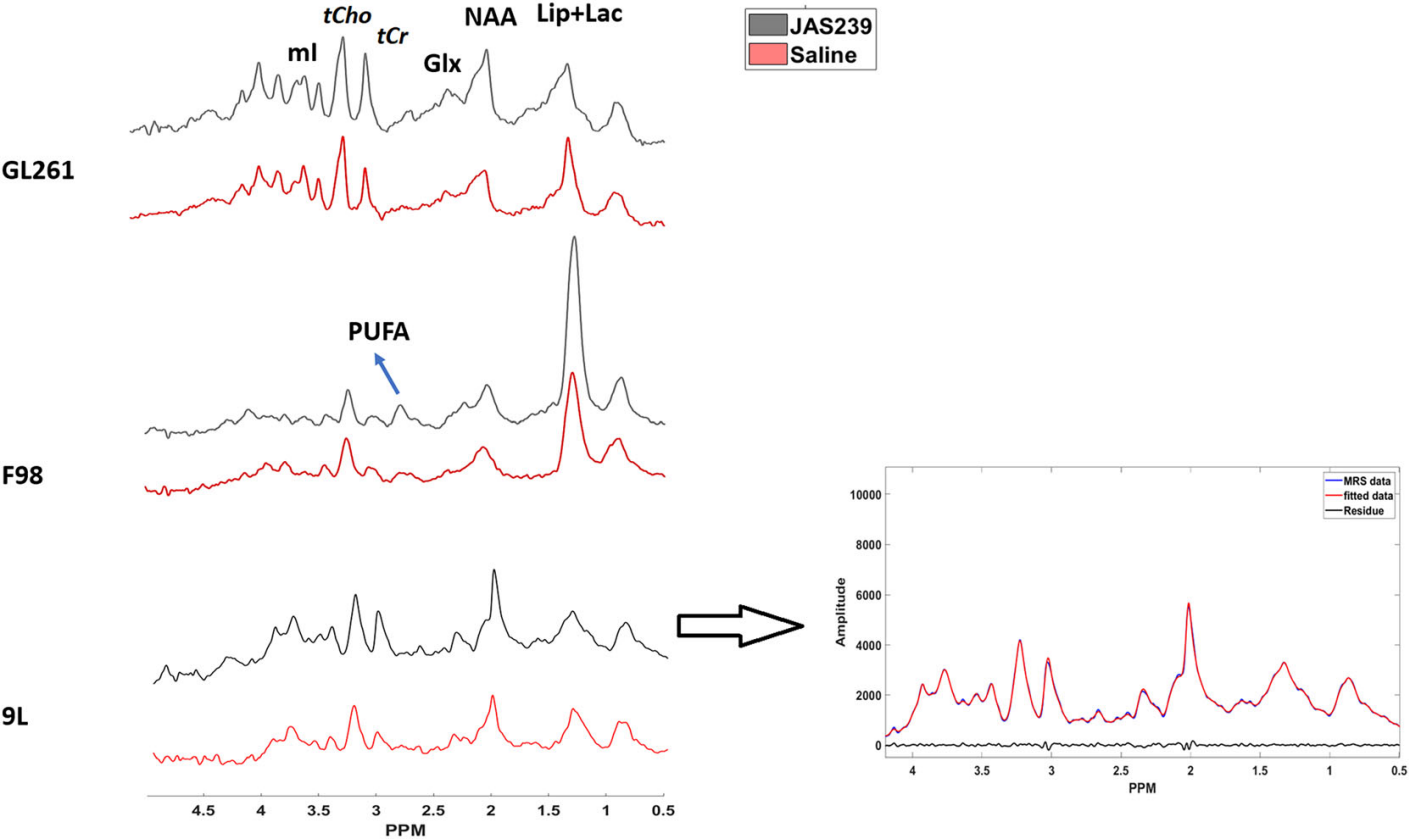
^1^H MR spectra comparing JAS239‐ and saline‐treated tumors showing Lip + Lac, NAA, Glx, tCr, tCho and mI peaks from the three tumor models. The presence of the PUFA peak was only observed in JAS239‐treated F98 tumors. The fitting of the above‐mentioned peaks in the spectra is shown on the right with the original spectra in blue, fitted spectra in red and residual in black.

The F98 tumors demonstrated a near significant increase in (Lip + Lac)/tCr ratio (97%, *p* = 0.052; Figure 6B) with treatment as compared with the untreated group at T6. On the other hand, 9L tumors demonstrated a decrease (−20%; Figure 6C) with treatment at T6, but this was not statistically significant. The (Lip + Lac)/tCr ratio in the GL261 model exhibited no significant differences after JAS239 treatment (Figure 6A) compared with the untreated group at T6. The presence of the polyunsaturated fatty acid (PUFA) resonance at 2.8 ppm, indicative of apoptosis, was also only observed in JAS239‐treated F98 tumors at T6 (Figure 7); however, due to higher CRLB values, this metabolite was not quantified.

**FIGURE 8:**
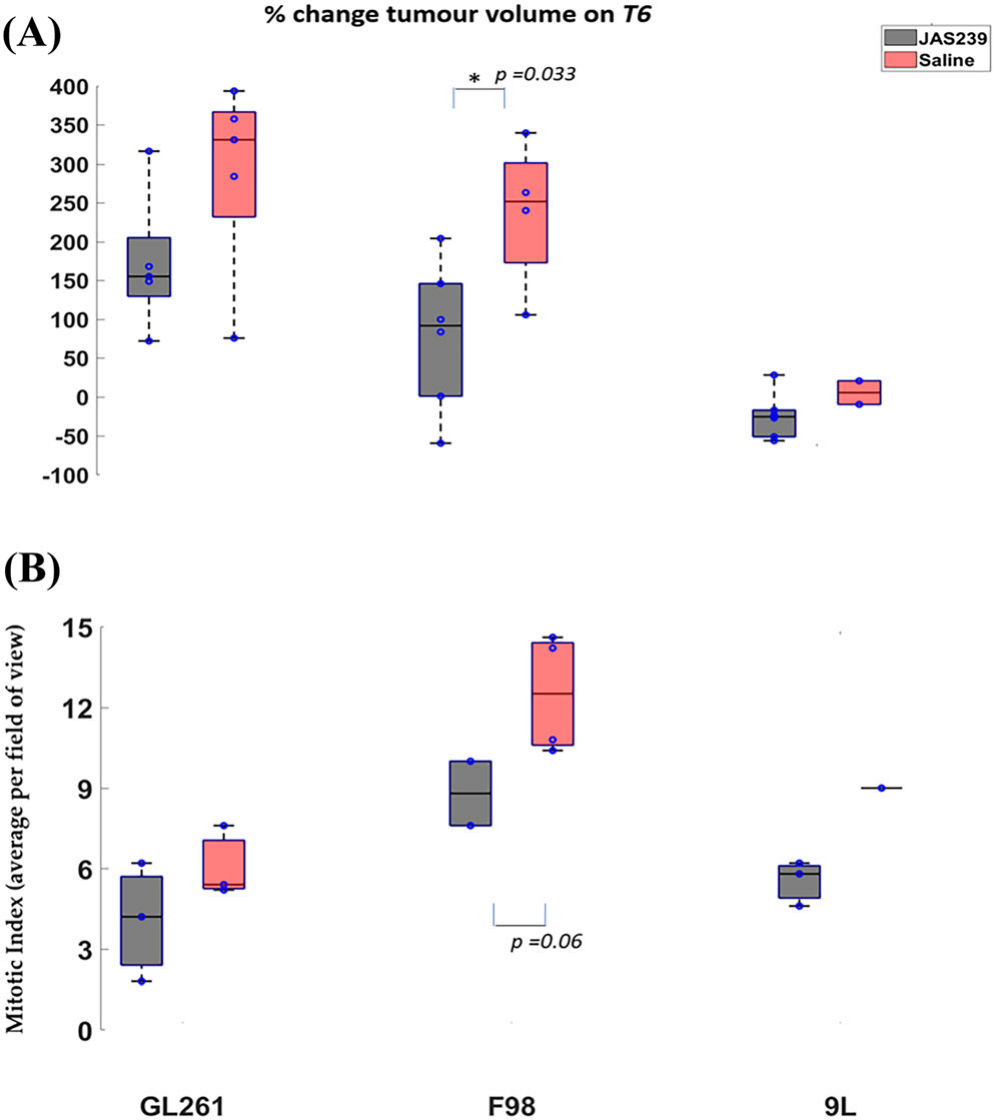
A, Box plots comparing percentage change (with respect to baseline) in tumor volume between JAS239 and control groups in GL261 mouse, F98 rat and 9L rat GBMs. B, Average mitotic index per HPF comparing JAS239 and saline control tumors in GL261 mouse, F98 rat and 9L rat GBMs. A single asterisk indicates that the difference between groups reached a significance level of 0.05.

**FIGURE 9:**
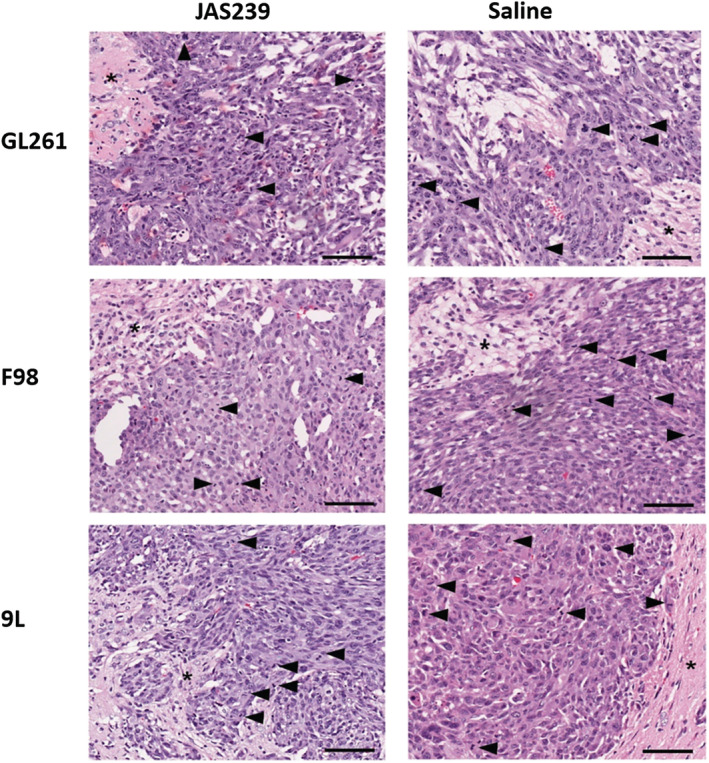
H&E images of rodent brain tumors of JAS239‐ and saline‐treated GL261 tumor, F98 tumor and 9L tumor. Arrow heads, mitotic bodies/nuclei; asterisk, normal brain parenchyma at tumor margin; scale bar, 100 μm.

In general, the GL261 and F98 tumors grew faster and were larger than the 9L tumors. Tumor volumes were determined and percentage change in volume was calculated from the baseline for all models (Figure 8A). Although treatment with JAS239 did not induce tumor regression in any of the models, F98 tumor growth was significantly reduced (*p* = 0.033) compared with the growth in control tumors, and growth arrest was also observed in both GL261 and 9L tumors, although it was not significant in comparison with untreated controls. Quantification of the mitotic index at the tumor margins (Figure 9) demonstrated a lower mitotic index in JAS239‐treated tumors across all models (Figure 8B), with the largest effect seen in F98 tumors; however, these reductions were not significant, most probably due to the smaller sample size, in either GL261 or 9L tumors.

## 4 DISCUSSION

In this study, we observed that JAS239 induced anti‐tumor activity and metabolic alterations in all intracranial GBM models tested. These results are promising in demonstrating JAS239's ability to cross the blood–brain barrier and penetrate the tumor mass, which is of particular importance when developing novel drugs targeting tumors of the brain. Furthermore, these data support cell metabolism as an effective anti‐cancer target, and the use of MRS as a non‐invasive methodology to assess treatment response overtime.

ChoK phosphorylates free choline into phosphatidylcholine (PC), and as a result increased levels of PC are considered a marker of proliferation and tumor progression in many tumor types.^34^, ^35^, ^36^ Specifically, increased ChoK expression has been shown to be sufficient to drive malignant transformation and is correlated with a higher tumor grade in patients with GBM.^37^, ^38^ Inhibition of ChoK has shown promising results in vivo across breast and GBM models via measurement of tCho, alterations of which are considered to be driven via changes in PC levels, using MRS.^19^, ^20^ In a recent study in F98 tumors, we reported a sustained decrease in permeability and perfusion and increased necrosis in response to the second‐generation ChoK inhibitor JAS239.^39^ Our study investigated JAS239 treatment on GBM metabolism, and demonstrated a trend of reduction in tCho concentration and tCho/tCr ratio in all GBM models treated with JAS239. The largest reduction was observed in the 9L model followed by the GL261 and F98 models. Similar results were observed for the tCho/NAA ratio. However, the effect of JAS239 on the reduction of tCho in these tumors was not as striking as previously reported for the ChoK inhibitor MN58b treatment of rat F98 GBMs^20^ or JAS239 treatment in a breast cancer model.^21^ Although a previous study^21^ reported more than 50% reduction in tCho with JAS239 treatment, it should be noted that it was performed on smaller, subcutaneous breast tumors. When we assessed the anti‐tumor activity of JAS239, we observed a significant growth arrest in F98 tumor volume (*p* = 0.033) and a trend of growth arrest in GL261 and 9L tumors. Although the largest percentage difference in tumor volume was demonstrated in the GL261 model, this was not significant because of the larger variation in data points in control animals. While the reductions in tCho were only modest, the JAS239‐induced reduction in tumor volume demonstrated that JAS239 is efficacious in these models and exhibits promising anti‐tumor activity. Furthermore, our experimental model tested JAS239 on large, well established intracranial tumors (>30 mm^3^) compared with the previous study,^21^ where JAS239 treatment was initiated three days after tumor cell inoculation and led to significant reductions in tumor volume. Additionally, the largest reductions in tCho concentration and tCho/tCr ratios were observed in the tumor regions, indicating that JAS239 selectively inhibits proliferating cells within the tumor, where ChoK is overexpressed, and not in the normal tissue. These data are further supported by cell cycle analysis in our laboratory, which showed a clear G0/G1 cell cycle arrest in these cells in response to JAS239 treatment (data not shown). To further explore the anti‐proliferative effect of JAS239 in these tumors we also quantified the mitotic index at the tumor periphery, where actively proliferating tumor cells reside. We observed a reduction in the mitotic index with JAS239 in all the tumor models, with a near significant reduction in the F98 tumors, supportive of JAS239's anti‐proliferative effect.

GBM is a highly metabolically active tumor with predominant metabolic changes, such as the upregulation of glycolysis and glutaminolysis with minimal oxidative phosphorylation of glucose by the TCA cycle.^6^, ^40^ The main advantage of increased glycolysis in cancer cells is not the efficient production of ATP, but instead the maintenance of pools of metabolites to feed pathways branching from glycolysis, including the pentose phosphate pathway for nucleotide biosynthesis, serine, Gly, one‐carbon metabolism and/or glycerol synthesis for lipid biosynthesis.^41^ However, the ‘Warburg effect’ is not a universal feature in all cancer cells. Both in vitro and in vivo studies have shown that cancer cells have functional mitochondria that can respire efficiently. Researchers have postulated that within a solid tumor, where there are generally hypoxic clusters, these cells may preferentially utilize glucose for glycolytic energy production (the Pasteur effect), whereas the highly proliferative cells on the tumor periphery, adjacent to blood vessels, may utilize alternative sources of fuel such as lactate and acetate for oxidative metabolism in combination with aerobic glycolysis.^5^, ^6^ Therefore, in addition to the inhibition of tCho, we also investigated other key metabolites associated with GBM growth and progression, such as glutamine, lactate and mI.

Interestingly, we observed a reduction in Glx in JAS239‐treated animals only in F98 tumors; while both 9L and GL261 demonstrated only modest reductions. This might be due to a reduction in glutamate. However, since standard PRESS sequences cannot differentiate between the overlapping resonances of glutamine and glutamate, it is possible that the reduction in the Glx peak observed in our study may also be due to changes in glutamine. These data demonstrate the metabolic heterogeneity of these GBM models with respect to Glx metabolism in response to JAS239. Heterogeneity in glutamine metabolism has previously been demonstrated across eight primary GBM models, both in vitro and in vivo, whereby different subtypes of GBM were identified and grouped as either ‘glutamine high’ or ‘glutamine low’.^42^ As expected, the inhibition of glutamine metabolism only affected proliferation and tumor development in the ‘glutamine‐high’ subgroup. Although the mechanism by which JAS239 affects Glx metabolism in F98 GBM tumors is unknown, this could be suggestive of F98 tumors being possibly susceptible to inhibition of glutamine–glutamate metabolism. Furthermore, alterations in the relevant transporters and enzymes involved in these metabolic pathways must be considered. In fact, a tissue microarray analysis of 209 human GBM patient samples showed that key enzymes involved in glutamine metabolism, such as glutamine synthetase (GS), varied dramatically between patients, and significant intra‐tumoral heterogeneity of GS expression was also evident.^43^

mI is another important metabolite in the brain, as it acts as an osmolyte and is essential in key signal transduction pathways.^44^, ^45^ It is an important substrate for the production of the phosphatidylinositol lipids and is characteristically increased in brain disorders, including brain malignancies.^13^, ^15^ We observed a significant reduction in the mI/tCr ratio in JAS239‐treated F98 tumors, and a non‐significant reduction in treated GL261 and 9L tumors. Increased mI levels have been correlated with glial cell proliferation.^45^ In the current study, a reduction in mI/tCr ratio in the treated animals indicates that JAS239 induced proliferation arrest or a switch in the cell cycle as mentioned above. One possible mechanism for this JAS239‐induced reduction in mI/tCr is via the cell cycle transcription factor E2F1. E2F1 has been shown to directly upregulate the expression of the enzyme ISYNA1, the rate‐limiting step in the synthesis of mI via glucose‐6‐phosphate; inhibition of E2F1 may reduce the synthesis of mI and impair GBM progression.^46^ We observed that JAS239 significantly reduces the protein expression of E2F1 in four GBM cell lines, with the most notable reduction in F98 cells (data not shown), potentially explaining this observation of JAS239‐induced reductions in mI/Cr levels. These changes could also be due to Gly, as at short echo times the Gly resonance overlaps with the mI signal at 3.56 ppm. In order to differentiate overlapping resonances, we have used a basis set that includes both mI and Gly peaks for fitting the resonance at 3.56 ppm. Additionally, since mI is present at much higher concentrations than the Gly level in vivo, we believe that most of the signal originates from the mI peak instead of Gly.

Lipids are essential water insoluble molecules that are vital for structural integrity and production of signaling molecules, and are a major source of energy.^47^ It is well documented that lipid metabolism is altered in cancer; rapidly proliferating cancer cells require increased phospholipid synthesis for cell membrane synthesis. Moreover, Lip + Lac resonances play a pivotal role in the diagnosis and monitoring of brain tumors as they are characteristically increased in high‐grade tumors such as GBM.^48^, ^49^, ^50^ Lactate is produced by tumor cells and is indicative of anaerobic metabolism and glycolysis, which are commonly associated with necrosis.^49^, ^51^ Lipid resonances arise from the methylene chains in mobile triglycerides and cholesterol esters, which often accumulate at sites of necrosis.^49^ Increased levels of Lip and Lac have been correlated with tumor grade and poor prognosis.^51^ Interestingly, of the three GBM models assessed, we observed a significant increase in the (Lip + Lac)/Cr ratio only in JAS239‐treated F98 tumors. This indicates that F98 tumors are more aggressive and necrotic, and have a glycolytic phenotype. Levels of lipids could also be altered due to increased apoptosis and cell death.^52^, ^53^ The 9L model exhibited the opposite effect, with a JAS239‐induced reduction in Lip + Lac. Furthermore, increased levels of PUFAs are typically observed in tumors after treatment and are indicative of increased apoptotic cell death.^52^, ^54^ MN58b treatment has shown to increase the 1.3 ppm Lip + Lac resonance in treated tumors along with increased PUFA resonance at 2.8 ppm indicating induction of apoptosis after treatment.^20^ Increased levels of lactate, lipids and PUFA whilst reductions in saturated fatty acid mobile lipids (1.3 ppm) and mI have been reported after temozolomide treatment in GL261 GBM, suggesting treatment‐induced apoptosis.^55^ In agreement, we also observed an increase in PUFA resonance, but only in the F98 JAS239‐treated tumor, supportive of JAS239 efficacy in this model. However, one needs to bear in mind that necrosis can account for a significant proportion of the tumor, particularly in the F98 GBM model. As a result, the spectrum can include these necrotic areas despite taking great care to include only viable portions of the tumor in the MRS voxel. In necrotic tumors, measurement of elevated lipids maybe more indicative of tumor progression than treatment‐induced changes in metabolites.

GBM inter‐ and intratumoral heterogeneity has been widely accepted based on genomic, proteomic, metabolomic and in vivo models. Therefore, one needs to be careful when choosing the most suitable preclinical model for the most translatable results. The models used in this study represent different phenotypic and genetic types of GBM and have all been used extensively in the MRI literature.^23^, ^24^, ^25^, ^26^ Furthermore, one of the main advantages of using the F98, GL261 and 9L models is that they can be implanted orthotopically in syngeneic, immune competent animals. More specifically, the 9L gliosarcoma model has been widely used to investigate mechanisms and development of drug resistance and transport of drugs across the blood–brain barrier.^24^ Whilst the 9L tumor is not considered an invasive model, it shares some histological similarities with GBM tumor cells such as S100B positive. Similar to human GBM cells, 9L cells have a mutant p53 gene and overexpress EGFR, which accounts for about 25% of human GBM tumors.^24^ On the other hand, F98 and GL261 tumors recapitulate the highly invasive and aggressive phenotype of human GBM. F98 tumors overexpress PDGFβ and Ras along with an increase in EGFR, cyclin D1 and cyclin D2, whilst GL261 tumors express stem cell marker CD133, which is associated with GBM transformation and increased resistance to environmental cues such as hypoxia.^24^, ^25^ Both F98 and GL261 tumors also present with characteristic hypoxic and pseudo‐palisading necrotic regions.

Hypoxia is a common feature of GBM and it tightly regulates many pathways involved in cell metabolism via hypoxia inducible transcription factors HIF‐1 and HIF‐2. For instance, HIF‐1 has been shown to regulate several steps of the glycolytic pathway^56^ and is also correlated with induced therapeutic resistance.^57^ A recent in vitro study investigated the effect of hypoxia and the ChoK inhibitor JAS239 on choline metabolism in brain tumor cells.^58^ The JAS239‐induced reduction in PC/GPC ratio was higher in hypoxic 9L cells, which are less aggressive GBM cell lines as compared with hypoxic F98 cells, indicating an interplay between hypoxia, invasion and choline metabolism.^37^, ^58^, ^59^ However, the degree of hypoxia is known to differ between GBM models and within a single tumor mass owing to tumor microenvironment heterogeneity.^60^ This could explain the marked variation in treatment response across GBM models as evidenced in our study across the three models tested. Since the 9L tumors are less aggressive and grow more slowly than F98 tumors, we initially hypothesized that these tumors will demonstrate greater therapeutic efficacy and significant changes in tCho; however, we observed a greater anti‐tumor effect of JAS239 in F98 and GL261 models. We demonstrated a heterogeneous metabolism across the three models, which was also reflected in the response to JAS239 treatment.

Some limitations and considerations of this study include the difficulties we experienced in consistently generating 9L tumors, as these tumors would either not grow or even self‐regress in some cases. For example, one saline‐treated 9L tumor demonstrated a decrease in tumor volume and in tCho. When the H&E stain from this tumor was evaluated, we found that the mass did not have any tumor cells but was rather composed of massive areas of inflammation, suggesting that the tumor self‐regressed (Figure S5). An additional limitation of the study was the relatively small window of study duration, as both the F98 and GL261 models were extremely aggressive, limiting the therapeutic window. Furthermore, the treatment dose of JAS239 was based on a previous study in mice with subcutaneously implanted breast cancer,^21^ and may not be the optimal dose. Finally, histology was performed 3 (in rats) and 5 (in mice) days after completion of JAS239 treatment, and it is possible that the tumors regrew during this time, masking treatment effects that may have been acute. However, as we analyzed the MRS data longitudinally during and at the end of treatment, we believe we have captured the metabolic changes induced by JAS239. Future studies should investigate these aspects along with an optimized JAS239 dose regimen, as a single drug regime or in combination with radiation and temozolomide, which are commonly used clinically for GBM treatment. Also, an intermediate or long *T*_E_ spectrum would have better described the contribution of Gly in describing the change in mI. However, we chose to use a short *T*_E_ spectrum for optimal SNR as well as being able to observe both short and long *T*_2_ metabolites. In order to differentiate overlapping resonances, we have used a basis set that includes both mI and Gly peaks for fitting the resonance at 3.56 ppm. Similarly, we relied on a basis set that includes both Lip and Lac peaks for fitting the resonance at 1.33 ppm. Finally, these data were generated in chemically induced rodent GBM cell lines that form large tumors quickly, and therefore extrapolation to the human context must be made with caution. One future consideration would be patient‐derived xenograft models, in which time to tumor formation and therapy resistance may more accurately represent human GBM.

## 5 CONCLUSION

MRS can be used to assess specific changes in GBM metabolism for a better understanding of cancer metabolism, specifically changes therein due to ChoK inhibition.

## CONFLICTS OF INTEREST

The authors declare that they have no conflict of interest. No external funding sources were involved in the design of the study; collection, analyses, or interpretation of data; writing of the manuscript; or decision to publish the results.

## Supporting information


**Figure S1:** Box plots comparing percentage change (with respect to baseline) in Cho/Water ratio between JAS239 and control groups in contralateral and tumor region in GL261 mouse GBM (A), F98 rat GBM (B) and 9L rat GBM (C). A single asterisk indicates that the difference between groups reached a significance level of 0.05, Figure S2: Box plots comparing percentage change (with respect to baseline) in mI/Water ratio between JAS239 and control groups in contralateral and tumor region in GL261 mouse GBM (A), F98 rat GBM (B) and 9L rat GBM (C). A single asterisk indicates that the difference between groups reached a significance level of 0.05, Figure S3: Box plots comparing percentage change (with respect to baseline) in Glx/Water ratio between JAS239 and control groups in contralateral and tumor region in GL261 mouse GBM (A), F98 rat GBM (B) and 9L rat GBM (C). A single asterisk indicates that the difference between groups reached a significance level of 0.05, Figure S4: Box plots comparing percentage change (with respect to baseline) in Lip+Lac/Water ratio between JAS239 and control groups in contralateral and tumor region in GL261 mouse GBM (A), F98 rat GBM (B) and 9L rat GBM (C). A single asterisk indicates that the difference between groups reached a significance level of 0.05, Figure S5: A) Representative H&E image of the 9L saline control rat which exhibited an inflammatory reaction to engrafted neoplasm is shown and the arrow/magnified section indicates a single cell identifiable as a neoplastic cell. B) MR spectra in the tumor region from the same rat before and after saline injections (T0 versus T6) showing decrease in total choline on T6. C) 9L non‐tumor with signs of inflammation D) 9L tumor with large neoplastic cells.
