# Supplementary material for: Metabolic changes in glioblastomas in response to choline kinase inhibition: In vivo MRS in rodent models
Source: NMR Biomed. 2022 Nov 10;36(3):e4855. doi: 10.1002/nbm.4855 (PMC10078495; doi:10.1002/nbm.4855)
Supplement: Supplementary file 1 — Figure S1: Box plots comparing percentage change (with respect to baseline) in Cho/Water ratio between JAS239 and control groups in contralateral and tumor region in GL261 mouse GBM (A), F98 rat GBM (B) and 9L rat GBM (C). A single asterisk indicates that the difference between groups reached a significance level of 0.05, Figure S2: Box plots comparing percentage change (with respect to baseline) in mI/Water ratio between JAS239 and control groups in contralateral and tumor region in GL261 mouse GBM (A), F98 rat GBM (B) and 9L rat GBM (C). A single asterisk indicates that the difference between groups reached a significance level of 0.05, Figure S3: Box plots comparing percentage change (with respect to baseline) in Glx/Water ratio between JAS239 and control groups in contralateral and tumor region in GL261 mouse GBM (A), F98 rat GBM (B) and 9L rat GBM (C). A single asterisk indicates that the difference between groups reached a significance level of 0.05, Figure S4: Box plots comparing percentage change (with respect to baseline) in Lip+Lac/Water ratio between JAS239 and control groups in contralateral and tumor region in GL261 mouse GBM (A), F98 rat GBM (B) and 9L rat GBM (C). A single asterisk indicates that the difference between groups reached a significance level of 0.05, Figure S5: A) Representative H&E image of the 9L saline control rat which exhibited an inflammatory reaction to engrafted neoplasm is shown and the arrow/magnified section indicates a single cell identifiable as a neoplastic cell. B) MR spectra in the tumor region from the same rat before and after saline injections (T0 versus T6) showing decrease in total choline on T6. C) 9L non‐tumor with signs of inflammation D) 9L tumor with large neoplastic cells. [file NBM-36-0-s001.docx]

**Supplementary file: Metabolic changes in glioblastomas in response to choline kinase inhibition: *in vivo* magnetic resonance spectroscopy in rodent models**

**Sourav Bhaduri ^1^**†**, Claire Louise Kelly1**†**, Clémentine Lesbats1,2, Jack Sharkey1, Lorenzo Ressel3, Soham Mukherjee1, Mark David Platt1, Edward J Delikatny4 and Harish Poptani1***

*1Centre for Preclinical Imaging, Department of Molecular and Clinical Cancer Medicine, University of Liverpool, Liverpool, UK 2Division of Radiotherapy and Imaging, The Institute of Cancer Research, London, United Kingdom*

*3Department of Veterinary Anatomy Physiology and Pathology, University of Liverpool, Leahurst Campus, Chester, UK 4Department of Radiology, Perelman School of Medicine, University of Pennsylvania, Philadelphia, United States of America*

† These authors contributed equally to this work.


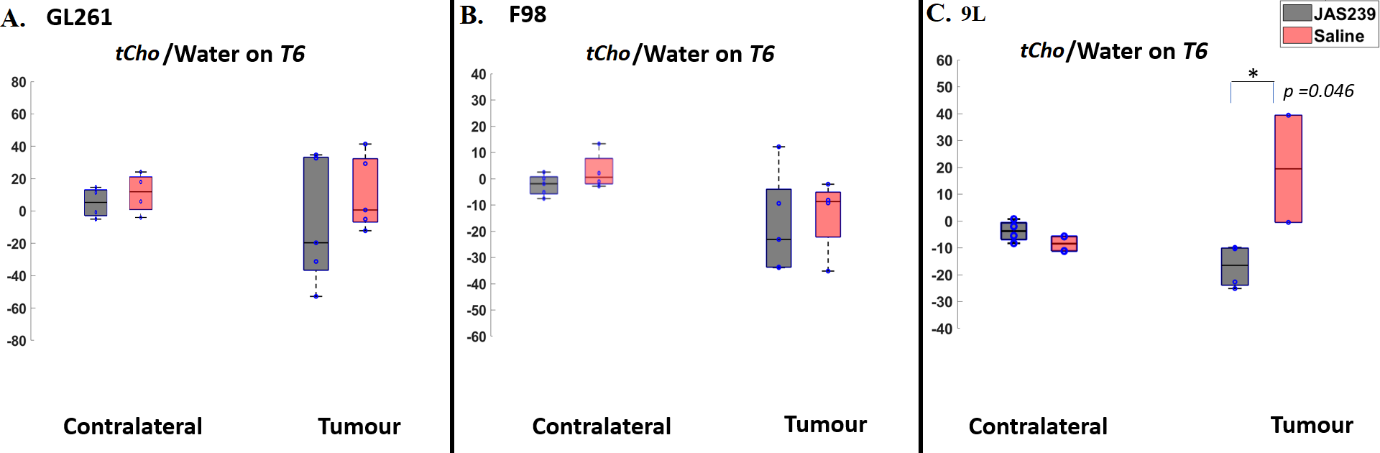


**Figure S1:** Box plots comparing percentage change (with respect to baseline) in Cho/Water ratio between JAS239 and control groups in contralateral and tumor region in GL261 mice GBM (A), F98 rat GBM (B) and 9L rat GBM (C). Single asterisk indicates difference between groups reached significance level of 0.05.


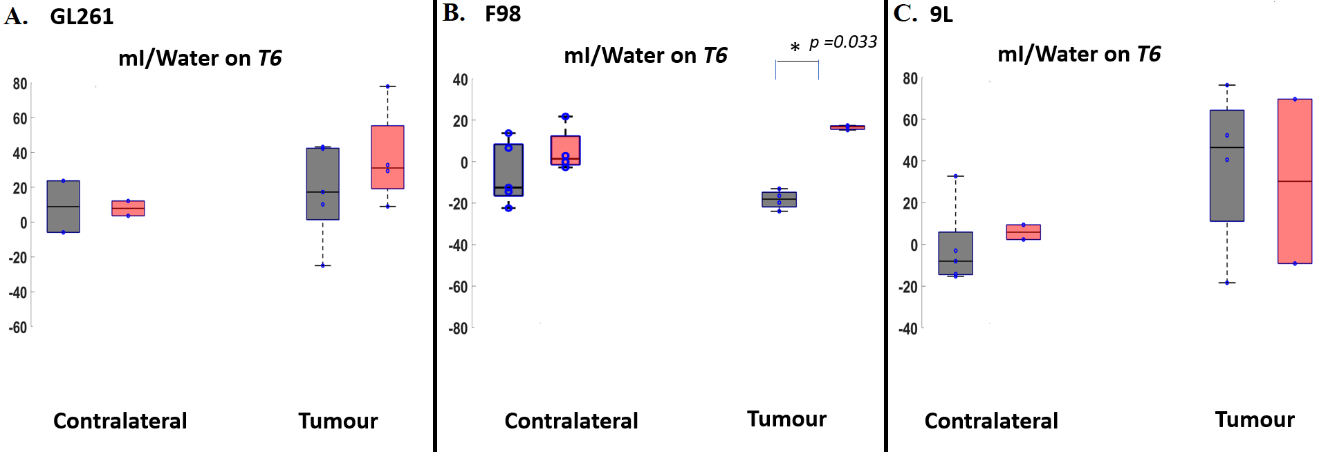


**Figure S2:** Box plots comparing percentage change (with respect to baseline) in mI/Water ratio between JAS239 and control groups in contralateral and tumor region in GL261 mice GBM (A), F98 rat GBM (B) and 9L rat GBM (C). Single asterisk indicates difference between groups reached significance level of 0.05.


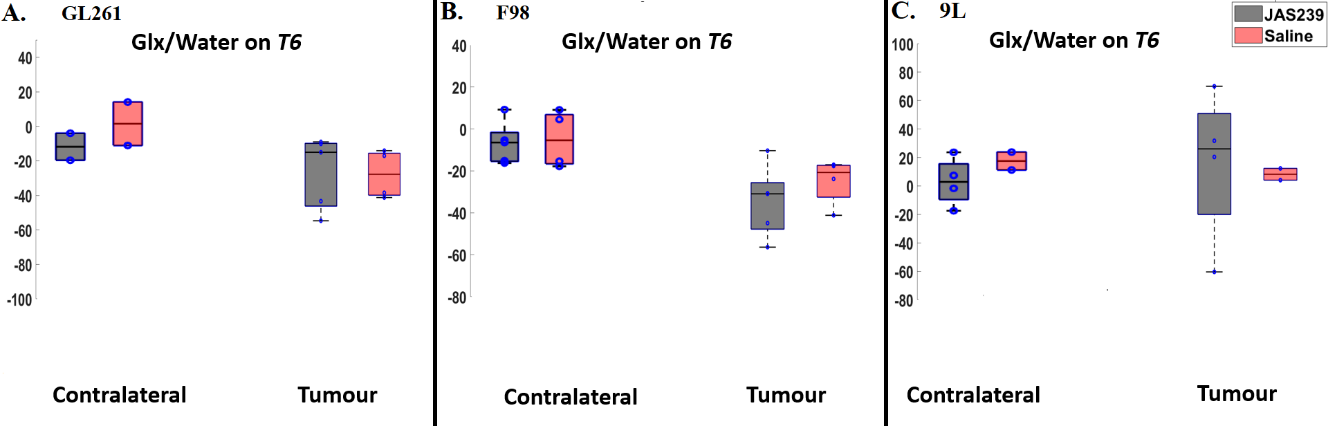


**Figure S3:** Box plots comparing percentage change (with respect to baseline) in Glx/Water ratio between JAS239 and control groups in contralateral and tumor region in GL261 mice GBM (A), F98 rat GBM (B) and 9L rat GBM (C). Single asterisk indicates difference between groups reached significance level of 0.05.


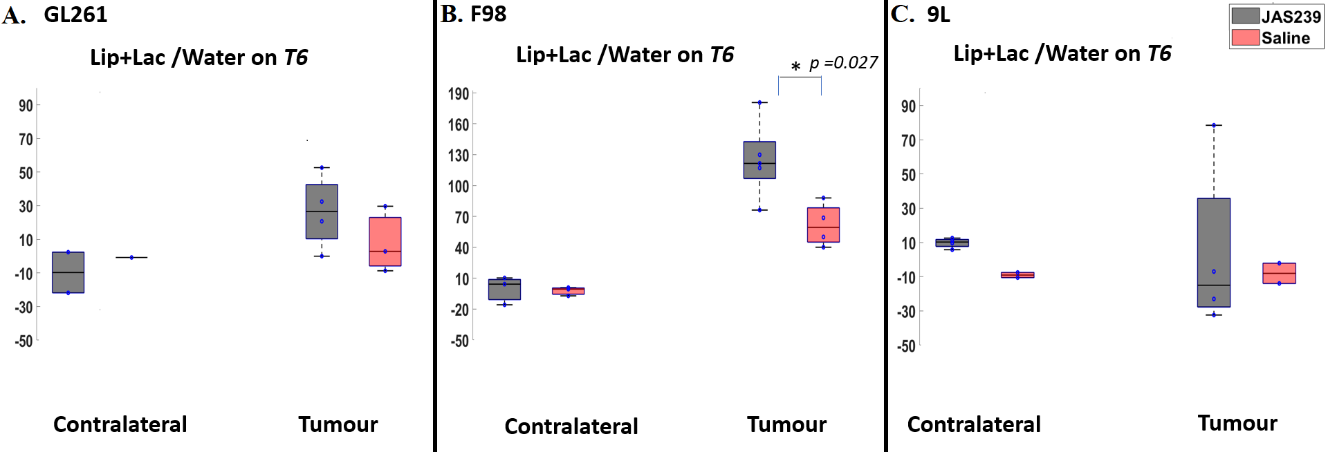


**Figure S4:** Box plots comparing percentage change (with respect to baseline) in Lip+Lac/Water ratio between JAS239 and control groups in contralateral and tumor region in GL261 mice GBM (A), F98 rat GBM (B) and 9L rat GBM (C). Single asterisk indicates difference between groups reached significance level of 0.05.


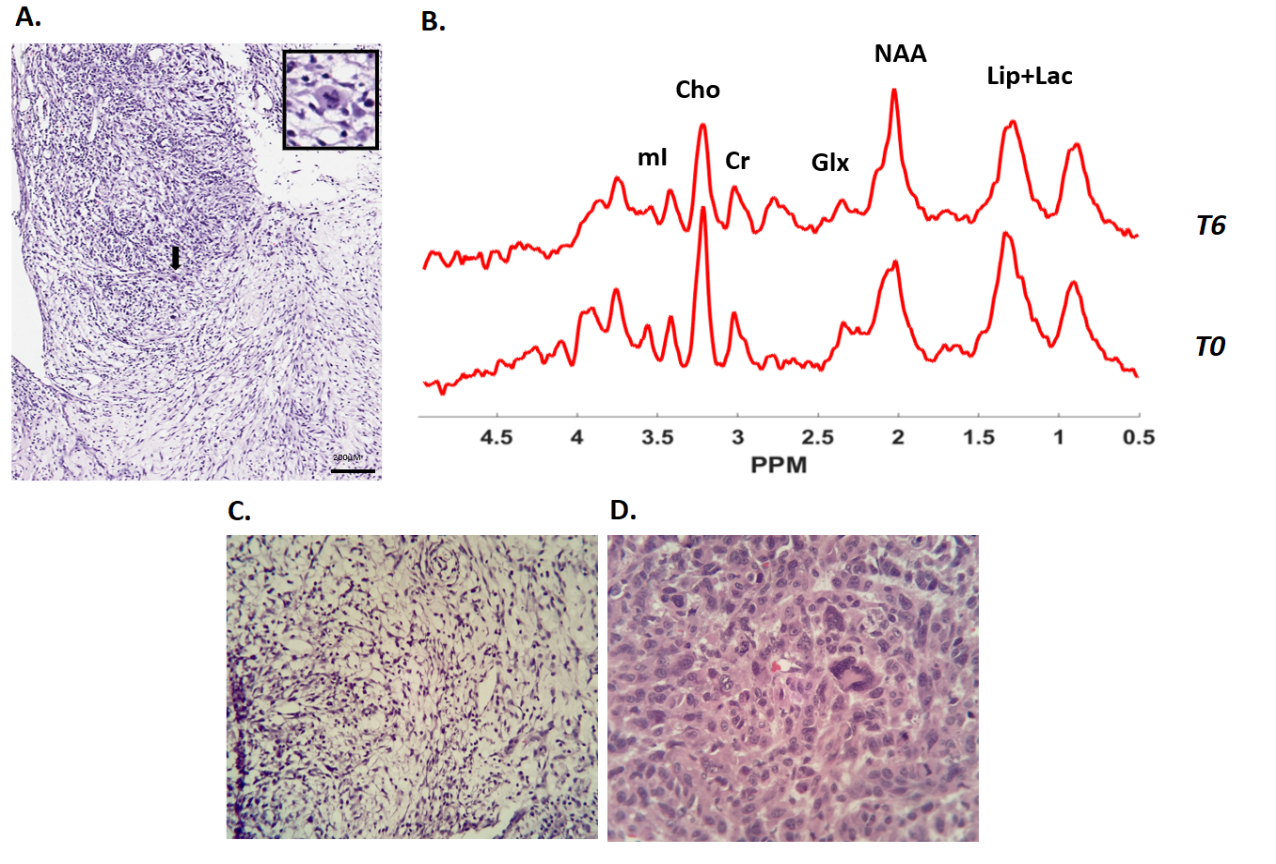


**Figure S5:** A) Representative image of the 9L saline control rat which exhibited an inflammatory reaction to engrafted neoplasm is shown and the arrow/magnified section indicates a single cell identifiable as a neoplastic cell. B) MR spectra in the tumor region from the same rat before and after saline injections (*T0* versus *T6*) showing decrease in total choline on *T6* C) 9L non-tumour with signs of inflammation D) 9L tumor with large neoplastic cells.
